# Ten reasons to screen women at risk of lung cancer

**DOI:** 10.1186/s13244-023-01512-8

**Published:** 2023-10-20

**Authors:** Marie-Pierre Revel, Guillaume Chassagnon

**Affiliations:** 1 Université Paris Cité, 85 Boulevard Saint-Germain, Paris, 75006 France; 2 Department of Radiology, Assistance publique des Hôpitaux de Paris, Hôpital Cochin, 27 Rue du Faubourg Saint-Jacques, Paris, 75014 France

**Keywords:** Lung cancer, Screening, Multidetector computed tomography

## Abstract

This opinion piece reviews major reasons for promoting lung cancer screening in at-risk women who are smokers or ex-smokers, from the age of 50. The epidemiology of lung cancer in European women is extremely worrying, with lung cancer mortality expected to surpass breast cancer mortality in most European countries. There are conflicting data as to whether women are at increased risk of developing lung cancer compared to men who have a similar tobacco exposure. The sharp increase in the incidence of lung cancer in women exceeds the increase in their smoking exposure which is in favor of greater susceptibility. Lung and breast cancer screening could be carried out simultaneously, as the screening ages largely coincide. In addition, lung cancer screening could be carried out every 2 years, as is the case for breast cancer screening, if the baseline CT scan is negative.

As well as detecting early curable lung cancer, screening can also detect coronary heart disease and osteoporosis induced by smoking. This enables preventive measures to be taken in addition to smoking cessation assistance, to reduce morbidity and mortality in the female population.

**Key points**

• The epidemiology of lung cancer in European women is very worrying.

• Lung cancer is becoming the leading cause of cancer mortality in European women.

• Women benefit greatly from screening in terms of reduced risk of death from lung cancer.

## Introduction

Lung cancer is the leading cause of cancer death worldwide [1]. After the onset of symptoms, most lung cancers are at an advanced stage when diagnosed and are not eligible for surgical resection which remains the cornerstone treatment when performed [2]. This is why lung cancer must be diagnosed at an early, preclinical stage through screening. The introduction of lung cancer screening in the US has induced a stage shift towards an early stage (stage I) at diagnosis, with improved survival [3]. Two large randomized trials have demonstrated that low-dose CT screening reduces lung cancer mortality. The risk of dying from lung cancer was reduced by 20% in the NLST trial, and by 24% in men and 33% in women in the NELSON trial [4, 5].

Results from smaller European studies, such as the MILD and LUSI trials, have also been positive, confirming a reduction in lung cancer mortality thanks to screening [6, 7].

At the end of 2022, scientific evidence led the European Council to update its 2003 recommendation on cancer screening to include lung cancer among the cancers to be screened. The Council encourages countries to “explore the feasibility and effectiveness of screening with use of low-dose computed tomography” [8]. The EU4Health program has funded the SOLACE (Strengthening the screening Of Lung cAnCer in Europe) project, to support Member States with the implementation of lung cancer screening.

With regards to its implementation, why is it important to include women at risk in screening??

## Reason # 1: “The incidence of lung cancer is rising steadily among European women”

As early as 2007, Levi et al. warned about the worrying epidemiological situation of lung cancer in young French and Spanish women with France having both the highest rate observed over the previous 3 decades and the highest increase over time in the last 2 decades [9]. The 2007 estimate was an incidence approaching 20/100,000 for the subsequent 2–3 decades in southern Europe. The KBP 2020 study confirmed this worrying trend for France. The study looked at 8999 patients from 82 French general hospitals diagnosed with lung cancer in 2020 and compared them with statistics from 2000 and 2010. The report showed that the proportion of women among lung cancer patients rose from 16% in 2000 to 24.3% in 2010 to 34.6% in 2020. The proportion of women diagnosed with lung cancer below the age of 50 was even 41% (8).

The statistics are just as worrying for many other countries. Fidler-Benaoudia et al. examined lung cancer incidence rates in young women versus young men in 40 countries across five continents. They compared age-specific lung cancer incidence from 1993–1997 through to 2008–2012. The incidence rate ratios between women and men increased significantly above unity in Canada, Denmark, Germany, New Zealand, the Netherlands, and the USA, with similar, albeit non-significant trends observed in 23 other countries [10].

The age-standardized incidence rates of lung cancer in most countries are projected to continue to increase dramatically by 2035, with peaks after the 2020s in most European, Eastern Asian, and Oceanian countries [11].

## Reason # 2: Lung cancer mortality is expected to surpass breast cancer mortality in European women

Carioli et al. predicted cancer mortality statistics for 2021 for the European Union (EU). The forecast for breast cancer in the EU was 13.3/100,000, corresponding to a reduction of 7.8%, while the forecast for lung cancer was 14.5/100,000, representing an increase of 6.5% [12].

Martin-Sanchez et al. calculated age-standardized mortality rates (ASMR) for lung and breast cancer from 2008 to 2014 with projections for the years 2015, 2020, 2025, and 2030 using a Bayesian model. In half of the 52 countries analyzed and in almost three-quarters of those classified as high-income countries, the ASMR for lung cancer has already exceeded or will exceed the ASMR for breast cancer before 2030 [13].

**Fig. 1:**
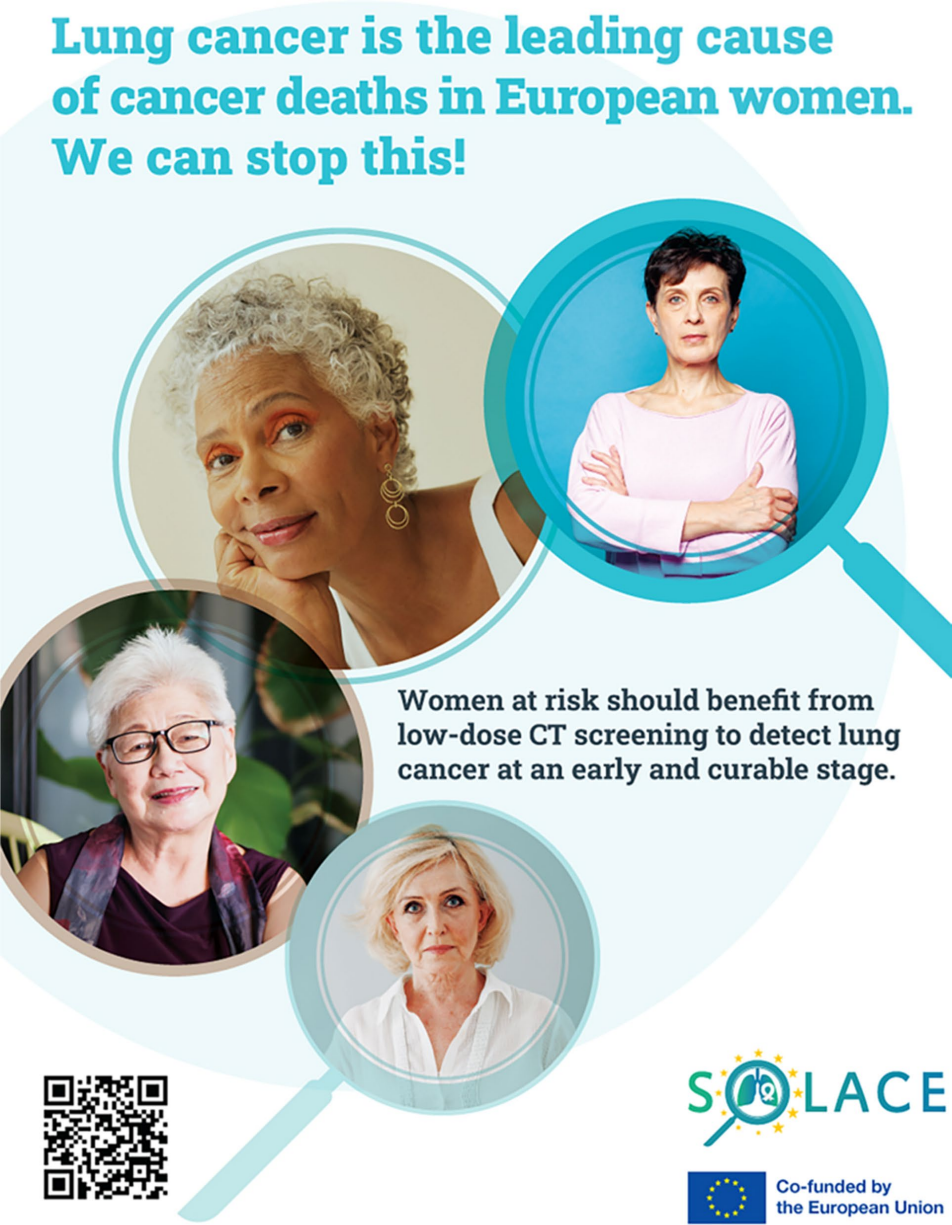
Poster used as part of the SOLACE project to promote women's participation in lung cancer screening

Cancer mortality forecasts for 2023 point to a 10% increase in lung cancer mortality among European women aged over 65 [14]. Lung cancer should not be the leading cause of cancer deaths among European women. It is with this message that the SOLACE project is promoting women’s participation in screening (Fig. 1).

## Reason # 3: To assess whether women’s risk of developing lung cancer is higher than men for the same level of smoking

Based on an analysis of 1889 cases, Zang et al. reported that dose–response odds ratios for cumulative smoking exposure were 1.2 to 1.7 times higher in women than in men for the three main histological types of lung cancer [15]. Risch et al. reported that for a history of 40 pack-years, the odds ratio was 27.9 for women and 9.60 for men [16].

There are, in fact, conflicting data concerning the higher risk of developing lung cancer in women on the basis of a tobacco exposure similar to that of men.

A multicenter case–control study conducted in Germany and Italy concluded that for comparable exposure to tobacco smoke, the risk of lung cancer was similar in women and men [17]. A systematic review and meta-analysis of 47 studies concluded that men had a higher susceptibility for cigarette smoking-attributable lung cancer than females [18].

However, an interim analysis of the CASCADE study [19] in France, exclusively recruiting female smokers (Fig. 1), estimated a prevalence of lung cancer (true positives) of between 2 and 3% (unpublished data). For an equivalent exposure to tobacco, this prevalence is more than 2 times higher than the 0.9% reported in the NELSON study, in which 83% of participants were male.

## Reason # 4: Women benefit the most from lung cancer screening in terms of lung cancer-related mortality

Greater reductions in lung cancer mortality were observed in women in the NELSON, NLST, and LUSI trials [4, 5, 7]. Women also showed a more pronounced reduction in lung cancer mortality with screening compared to men in the ITALUNG screening trial (41% versus 19%), although the difference was not statistically significant [20].

A recent Cochrane review pooled the results of four trials that reported lung cancer mortality risk reduction by gender [21]. Based on 26,965 participants in the NLST, NELSON, LUSI, and UKLS trials, screening reduced the risk of lung cancer mortality in women by 29% (RR 0.71, 95% CI 0.59 to 0.86) [4, 5, 7, 22]. In comparison, lung cancer mortality in men was reduced by 15% with low-dose CT screening based on the results of the same four trials and those of the DANTE trial [4, 5, 7, 22, 23].

## Reason # 5: Women have been under-represented in most lung cancer screening studies

Most studies included a majority of male participants, and the last sentence of the NELSON trial was “More research is required in women” [5].

As a result, there is a lack of data characteristics in women in lung cancer screening. Published data includes both men and women, and it is not possible to extract the characteristics of the cancers screened in women, in terms of the stage and histology. Similarly, it is not possible to extract data on the age and exposure to tobacco in women with positive screening, the rate of overdiagnosis, the radiation dose received, the psychological impact of screening, and its influence on smoking cessation in women.

However, female under-representation varied from study to study.

The Dante trial did not include any women, whereas the proportion of women in the NELSON trial was only 17% [5, 24]. Women represented 25% of participants in the screen arm in the UKLS trial, 29% of such participants in the French trial DEPISCAN, 32% in the MILD trial, and 44% in the Danish Randomized Lung Cancer CT Screening Trial [22, 25–27].

## Reason # 6: Screening can be used as an opportunity for smoking cessation, thus reducing the prevalence of female smoking

The evidence from controlled trials suggests that participating in lung screening significantly increases smoking cessation rates compared with the general population.

In DLCST, ex-smoker rates significantly increased from 24% at baseline to 37% at year 5 of screening [28]. Although there was no statistically significant difference in annual smoking status between the CT group and control group, the authors concluded that overall the screening program promoted smoking cessation. In the UKLS trial, the smoking cessation rates within the 2 years after recruitment were 21% and 24% in the control and screen arm, respectively [29]. In France, among 18–75-year-olds, male smoking prevalence declined from 60% in 1976 to 38% in 2010, whereas rates of smoking among women changed very little, approximately 30% throughout [30]. More recently, the proportion of women smoking daily in France has increased from 21% in 2019 to 23% in 2021, according to Santé publique France [31].

Thus, screening the female smoking population may therefore be one way of combating the rising prevalence of female smoking.

## Reason # 7: From an organizational point of view, lung cancer screening could be combined with breast cancer screening

Both lung and breast cancer screening are based on imaging equipment, enabling them to be carried out in a single location. Age ranges for lung and breast cancer screening largely coincide. Most lung cancer screening studies have included participants aged between 50 and 75 [32]. Recently, the US Preventive Services Task Force (USPSTF) updated its 2013 recommendation to extend lung cancer screening up to the age of 80 [33]. The justification was to increase the relative percentage of persons eligible for screening by 80% in men and by 96% in women.

For the average-risk women, most of the breast cancer screening guidelines recommend mammographic screening for those aged between 40 and 74 years, and specifically those aged 50–69 years who are regarded as the optimal age group for screening [34].

The MILD study compared annual and biennial lung cancer screening and showed similar lung cancer mortality. So, as far as screening intervals are concerned, it is possible for lung cancer and breast cancer screening intervals to coincide if the baseline low-dose CT scan is negative [26].

One way of inviting women for lung cancer screening in the CASCADE study is to include a flyer with information on the CASCADE study in the breast cancer screening invitation letter. Among the different invitation methods for screening, the combination of the flyer attached to the breast cancer screening invitation letter was the second most effective method for participation, after audio communication.

## Reason # 8: Adherence to lung cancer screening is particularly good among females who can be role models for others

Factors determining adherence to lung cancer screening have been evaluated in several studies. One study conducted in Spain reported that adherence to lung cancer screening was particularly good among females [35]. Another study reported higher adherence among women than among men screened through a decentralized program (39.2% versus 32.3%) [36]. Another study reported that female patients showed trends towards better adherence although not statistically significant [37]. Female sex and motivation were parameters associated with higher screening adherence rates in the study by Zulueta et al. [38]. Women who are willing to take part in screening can persuade their partners and other family members at risk to undergo screening as well.

## Reason # 9: Lung cancer screening can be an opportunity to screen for osteoporosis

Smoking is a recognized risk factor for postmenopausal osteoporosis [39].

Osteoporosis is a prevalent and treatable condition, but it remains underdiagnosed.

Taking the opportunity of lung cancer screening to analyze the thoracic vertebrae is extremely relevant, as vertebral fractures and bone density were independently associated with all-cause mortality among lung cancer screening trial participants [40].

Artificial intelligence has been evaluated for the automated assessment of osteoporosis. Some authors have trained a deep learning model for the detection of osteoporosis on low-dose CT scans performed for lung cancer screening and reported an AUC of 0.927 [41]. Automatic segmentation and radiomic texture analysis have also been used for this purpose [42].

Detection of osteoporosis is one of the secondary objectives of the CASCADE study along with assessment of coronary artery calcium score [19].

## Reason # 10: Lung cancer screening can be an opportunity to detect coronary heart disease in women

**Fig. 2:**
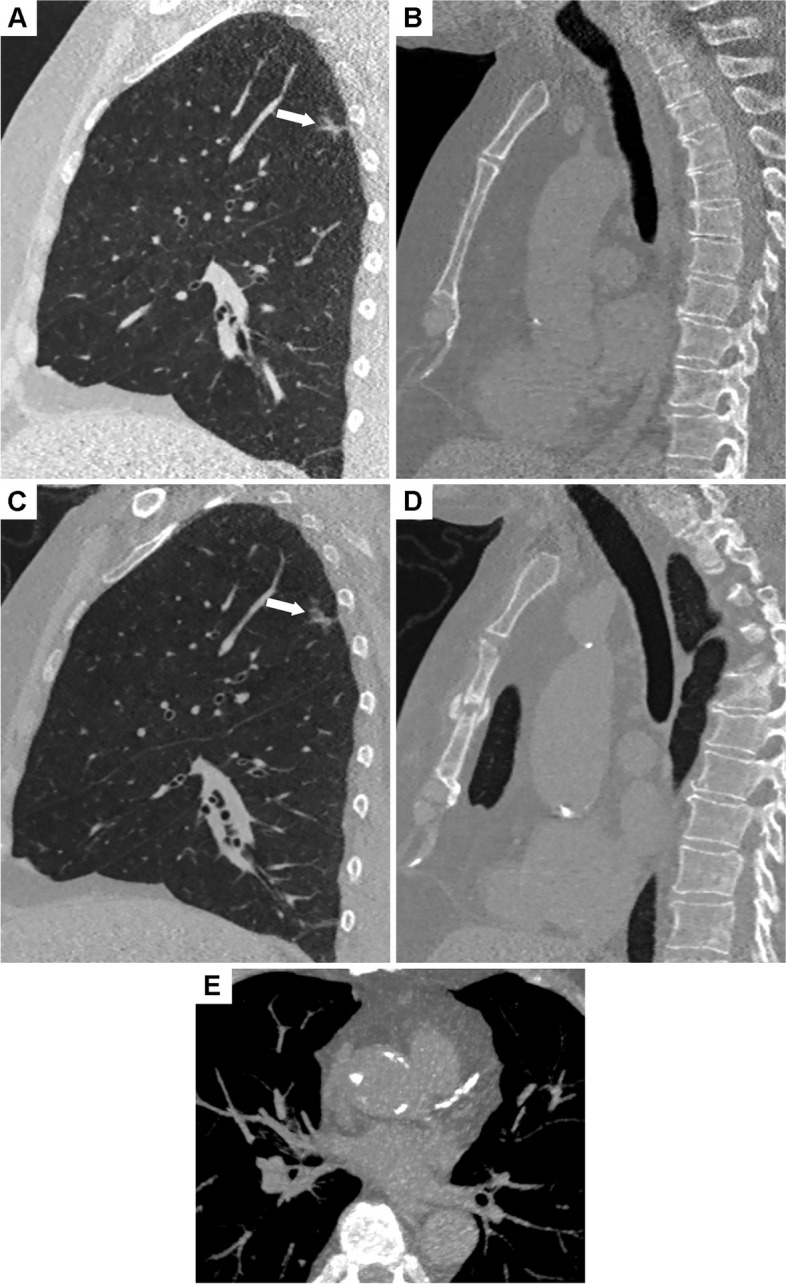
A sagittal reformat of a baseline CT scan in a 61-year-old participant shows an irregular subsolid nodule in the right upper lobe (arrow) (**A**), with the sternum showing no abnormalities (**B**). CT follow-up at 3 months (**C**) showed no change in the morphologically suspicious nodule (arrow), but a recent fracture of the sternum (**D**). This was due to cardiac massage for a cardiac arrest secondary to a myocardial infarction which occurred in the interval between the two CT scans. On the baseline CT, the left descending coronary artery was heavily calcified (**E**). A segmentectomy was performed after recovery, revealing an invasive acinar adenocarcinoma staged pT1a No Mo

The diagnosis of coronary heart disease is more difficult in women than in men. Gender differences in the clinical presentation of ischemic heart disease may contribute to this difficulty [43]. In addition, in the Framingham Heart Study, almost two-thirds of sudden deaths due to coronary heart disease in women occurred with no previous symptoms. Detecting a high calcium score during lung cancer screening may help prevent cardiac death. The reference technique for coronary calcium score analysis is based on the Agastston score which is calculated on a CT scan performed with cardiac synchronization [44]. However, visual ranking of coronary artery calcifications on low-dose CT is reliable for predicting Agatston score rank categorization [45]. Visual assessment of coronary artery calcifications on low-dose CT scans provides clinically relevant quantitative information on cardiovascular mortality, as reported by Shemesh et al. [46]. Figure 2 illustrates the relevance of this approach. Incidental aortic valve calcification may also be identified on non-gated thoracic CT. Of note, women may experience severe aortic stenosis at lower AVC scores than men [47].

## Conclusion

There are at least 10 excellent reasons to screen at-risk women for lung cancer, and of course, this does not mean that at-risk men should not be screened. These reasons are (i) scientific, as we lack data on women’s lung cancer screening; (ii) strategic, as women benefit greatly from screening at a time when lung cancer mortality among European women continues to rise; and (iii) organizational, as lung cancer screening can be combined with mammography screening.

In addition to all these reasons, women can contribute to smoking prevention by encouraging their partners and children not to smoke or to stop smoking, as women are more often concerned about the health of others than their own. This could help build a new tobacco-free generation.

Stopping tobacco consumption and production is the goal we need to reach if we are to relegate lung cancer to what it was before the cigarette era: an uncommon disease, and not the leading, albeit preventable, cause of cancer deaths.

## Data Availability

Not applicable.

## Abbreviations

ASMR: Age-standardized mortality rates
DLCST: Danish Randomized Lung Cancer CT Screening Trial
EU: European Union
LUSI: LUng cancer Screening Intervention
MILD: Multicentric Italian Lung Detection
NELSON: NEderlands–Leuvens Longkanker Screenings Onderzoek
NLST: National Lung Screening Trial
SOLACE: Strengthening the screening Of Lung cAnCer in Europe
UKLS: UK Lung cancer Screening
USPSTF: US Preventive Services Task Force
