# Correction: Ten reasons to screen women at risk of lung cancer

**DOI:** 10.1186/s13244-023-01570-y

**Published:** 2023-11-22

**Authors:** Marie-Pierre Revel, Guillaume Chassagnon

**Affiliations:** 1 Université Paris Cité, 85 Boulevard Saint-Germain, 75006 Paris, France; 2 Department of Radiology, Assistance Publique Des Hôpitaux de Paris, Hôpital Cochin, 27 Rue du Faubourg Saint-Jacques, 75014 Paris, France

**Correction: Insights into Imaging 14, 176 (2023)**

**https://doi.org/10.1186/s13244-023-01512-8**

Figure 1 of the originally published article [1] showed an incomplete figure legend. The correct figure legend should read:

"Poster used as part of the SOLACE project to promote women's participation in lung cancer screening.

The SOLACE project is co-funded under the EU4Health Programme 2021–2027 under grant agreement no. 101101187. Views and opinions expressed are however those of the author(s) only and do not necessarily reflect those of the European Union or HaDEA. Neither the European Union nor the granting authority can be held responsible for them."
